# Larval habitats, species composition and distribution of malaria vectors in regions with autochthonous and imported malaria in Roraima state, Brazil

**DOI:** 10.1186/s12936-021-04033-1

**Published:** 2022-01-14

**Authors:** Nathália Coelho Vargas de Almeida, Jaime Louzada, Maycon Sebastião Alberto Santos Neves, Thiago M. Carvalho, Júlio Castro-Alves, Teresa Fernandes Silva-do-Nascimento, Ananias A. Escalante, Joseli Oliveira-Ferreira

**Affiliations:** 1 Laboratório de Imunoparasitologia, Instituto Oswaldo Cruz, Fundação Oswaldo Cruz, Rio de Janeiro, Brasil; 2 Universidade Federal de Roraima, Boa Vista, Roraima Brasil; 3 Laboratório de Mosquitos Transmissores de Hematozoários, Instituto Oswaldo Cruz, Fundação Oswaldo Cruz, Rio de Janeiro, Brasil; 4 Instituto Nacional de Infectologia Evandro Chagas, Fundação Oswaldo Cruz, Rio de Janeiro, Brasil; 5 Department of Biology/Institute for Genomics and Evolutionary Medicine (iGEM), Temple University, Philadelphia, PA USA

**Keywords:** Malaria vectors, Larval habitats, *Anopheles darlingi*, *Anopheles albitarsis s.l.*, Human biting rate, Imported malaria

## Abstract

**Background:**

Malaria control requires local action. Assessing the vector diversity and abundance provides information on the local malariogenic potential or risk of transmission. This study aimed to determine the *Anopheles* species composition, habitats, seasonal occurrence, and distribution in areas with autochthonous and imported malaria cases in Roraima State.

**Methods:**

A longitudinal study was conducted from January 2017 to October 2018, sampling larvae and adult mosquitoes in three municipalities of Roraima State: Boa Vista, Pacaraima and São João da Baliza. These areas have different risks of malaria importation. Four to six mosquito larval habitats were selected for larval sampling at each municipality, along with two additional sites for adult mosquito collection. All larval habitats were surveyed every two months using a standardized larval sampling methodology and MosqTent for adult mosquitoes.

**Results:**

A total of 544 *Anopheles* larvae and 1488 adult mosquitoes were collected from the three municipalities studied. Although the species abundance differed between municipalities, the larvae of *Anopheles albitarsis s.l.*, *Anopheles nuneztovari s.l.* and *Anopheles triannulatus s.l.* were collected from all larval habitats studied while *Anopheles darlingi* were collected only from Boa Vista and São João da Baliza. Adults of 11 species of the genus *Anopheles* were collected, and the predominant species in Boa Vista was *An. albitarsis* (88.2%) followed by *An. darlingi* (6.9%), while in São João da Baliza, *An. darlingi* (85.6%) was the most predominant species followed by *An. albitarsis s.l.* (9.2%). In contrast, the most abundant species in Pacaraima was *Anopheles braziliensis* (62%), followed by *Anopheles peryassui* (18%). Overall, the majority of anophelines exhibited greater extradomicile than peridomicile-biting preference. *Anopheles darlingi* was the only species found indoors. Variability in biting times was observed among species and municipalities.

**Conclusion:**

This study revealed the composition of anopheline species and habitats in Boa Vista, Pacaraima and São João da Baliza. The species sampled differed in their behaviour with only *An. darlingi* being found indoors. *Anopheles darlingi* appeared to be the most important vector in São João da Baliza, an area of autochthonous malaria, and *An. albitarsis s.l.* and *An. braziliensis* in areas of low transmission, although there were increasing reports of imported malaria. Understanding the diversity of vector species and their ecology is essential for designing effective vector control strategies for these municipalities.

**Supplementary Information:**

The online version contains supplementary material available at 10.1186/s12936-021-04033-1.

## Background

Malaria incidence in the Brazilian Amazon reduced from 333,461 cases in 2010 to 156,916 in 2019 [1]. Regardless of such regional progress, control interventions have not been successful in states sharing international borders, where malaria cases continue to increase [2–4]. Roraima is a Brazilian state in the extreme north of the country that shares international borders with Guyana and Venezuela. Since 2016, this region has been experiencing an unprecedented flow of migrants from Venezuela due to that country's economic, social and political crises [3, 5–7]. This uncontrolled migration translates into a larger number of imported cases [2, 8].

Data from the Ministry of Health, Epidemiological Surveillance Information System—malaria (SIVEP-malaria), shows that the number of cases reported in Roraima almost tripled from 8969 in 2016 to 23,369 in 2018. Of those, 34% were imported cases from other states in Brazil (3,630 cases) and other countries (5513 cases) [1, 2], particularly from Venezuela (4478 cases) and Guyana (610 cases) [1]. Two municipalities in Roraima, Boa Vista (the capital) and Pacaraima (bordering Venezuela), reported more than half of the imported malaria cases in Brazil across international borders between 2007 and 2018. Although these municipalities are considered areas of low transmission risk, the influx of migrants and cases makes them vulnerable to malaria outbreaks and epidemics [2, 8]. The municipality of São João da Baliza (SJB) is located in the south of the state of Roraima, a region of tropical rainforest, endemic for malaria. In 2019, 634 cases were reported, of which 310 were acquired in the urban area of the municipality [1]. Furthermore, in Roraima state, *Plasmodium falciparum* infections were responsible for 9.8% of the autochthonous cases and 26% of the imported cases from other countries. Cross-border and authochtonous falciparum malaria threatens the Brazilian plan to eliminate this parasite from the country [2, 8], fuelling concern about importing parasites that are resistant to the drugs used to treat malaria in Brazil [9–12]. Therefore, characterizing the vector species involved in local transmission in these border municipalities and their larval habitats is considered a priority.

*Anopheles darlingi*, the most important malaria vector in the Brazilian Amazon is highly anthropophilic compared to other Amazonian anophelines, transmitting both *P. falciparum* and *Plasmodium vivax* [13–17]. *Anopheles darlingi* are found in a wide variety of larval habitats, such as streams, margin rivers, flooding areas, and dams surrounded by vegetation [14, 18–20]. Besides *An. darlingi*, other malaria vectors in the northern region include *Anopheles albitarsis s.l., Anopheles nuneztovari s.l., Anopheles triannulatus s.l.* and *Anopheles braziliensis*, which are mostly of local importance [4, 21–23].

In Roraima, previous studies conducted in Boa Vista reported that *An. darlingi* and *An. albitarsis s.l.* were the most relevant local vectors [24–27]. *Anopheles darlingi* had the highest infection rate, followed that of *An. albitarsis s.l.*, *An. braziliensis* and *An. nuneztovari s.l. Anopheles albitarsis s.l.* has been reported as the most common anopheline with the highest biting rate [22, 25–27]. Although *An. darlingi* and *An. albitarsis s.l*. are considered the primary malaria vectors in Boa Vista [24–27], *An. albitarsis s.l.* constitutes a complex of at least 10 species that are widely distributed in South America [25, 28–32]. However, only four species were incriminated as a vector of malaria parasites: *Anopheles deaneorum* [33] *Anopheles janconnae* (= *An. albitarsis* E)[26], *Anopheles marajoara* [34–36] and *Anopheles albitarsis* F [32, 37]. *Anopheles albitarsis* E (= *An. janconnae*) has been reported as a malaria vector in Boa Vista. This species was abundant in the savannah biome, anthropophilic and showed biting behaviour predominantly in the early evening [26].

Considering the ongoing high levels of imported malaria in Roraima [1, 2], and the diverse ecological landscape in the state [38], this study aimed to identify anopheline larval habitats and mosquito species distribution and behaviour in areas with imported (Boa Vista and Pacaraima) and autochthonous malaria (São João da Baliza). The identification of potential larval habitats and the ecological conditions that determine the presence of *Anopheles* species competent to transmit *Plasmodium* is an important contribution for the control and elimination of malaria in Roraima.

This information will allow for the assessment of potential local malaria vectors in each municipality and may explain differences in transmission patterns. The long-term goal is to direct vector control measures as part of integrated policies to reduce the region's malariogenic potential and mitigate, if possible, the effect of imported malaria cases.

## Methods

### Study areas

**Fig. 1:**
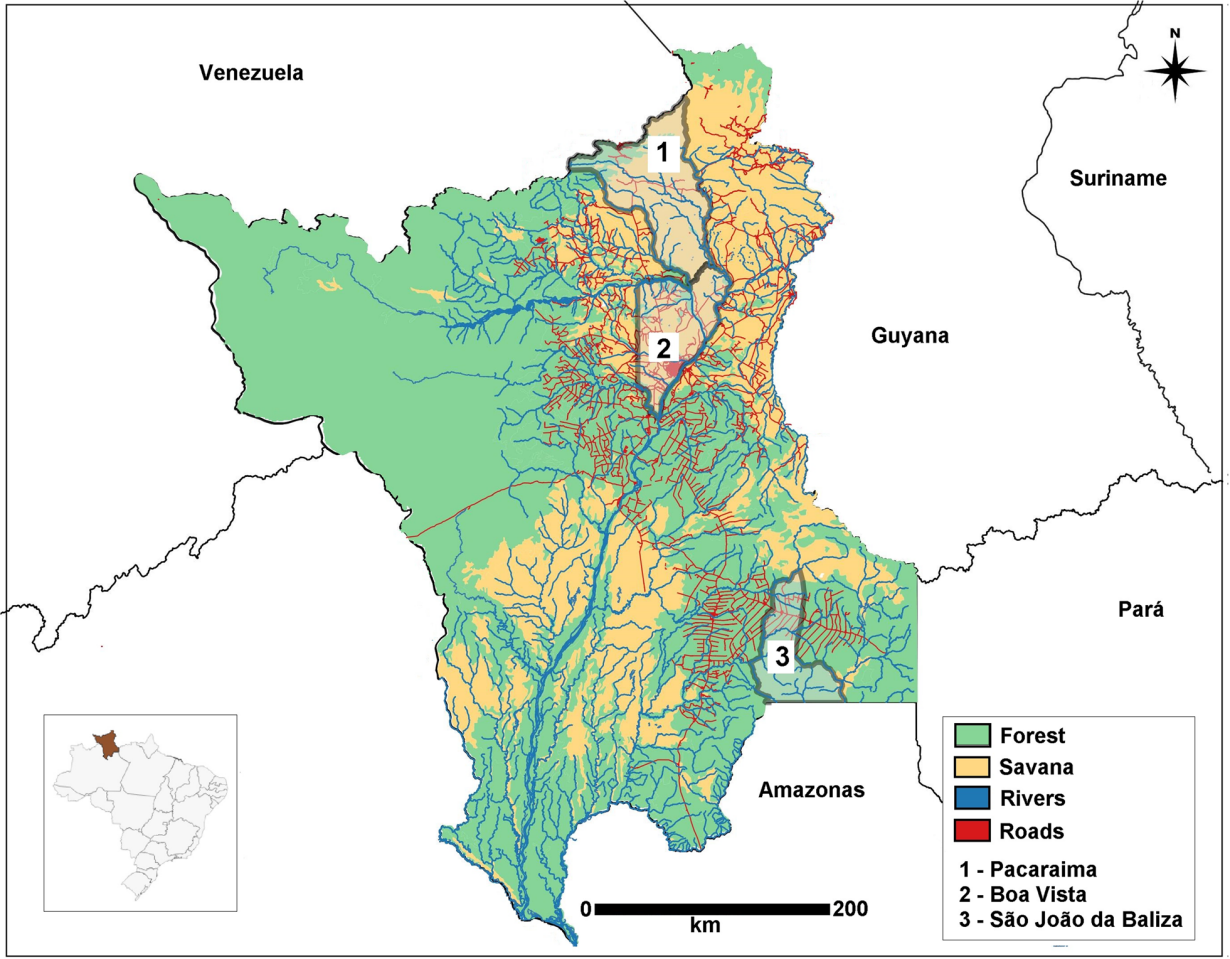
Map of Roraima State with highlights on rivers, roads, vegetation, and locations of the municipalities of Pacaraima—1, Boa Vista—2 and São João da Baliza – 3

The study was conducted in the municipalities of Boa Vista, Pacaraima and São João da Baliza, Roraima State (Fig. 1). These three municipalities were chosen because of their numbers of autochthonous and imported malaria cases, and their status in annual parasite incidence (IPA), a risk indicator for malaria transmission used by the Ministry of Health in Brazil. This indicator expresses the number of confirmed new infections, considering the likely place of infection, per year per 1,000 inhabitants. The urban areas of Boa Vista had a very low risk (IPA 0.4), while Pacaraima had a low risk (IPA 1.0) and São João da Baliza was considered a region with medium risk (IPA 37.8) for the year 2018. Boa Vista had an estimated population of 399,213 in 2019 and is located in the centre of the state (02°49′12" N; 60°40′23" W). It is 231 km from the main Venezuelan town in the country's malaria-endemic region (Santa Elena do Uairén), and it is 133 km from Guyana (Lethem). Its location allows easy access to the international borders and the state of Amazonas by highways. Indeed, Boa Vista predominated as the municipality with the most reported cases imported from neighbouring countries. In 2018, it registered 5,545 cases. Of these, 168 were autochthonous, 2115 were imported from Venezuela, and 533 from Guyana. *Plasmodium falciparum* infection imported from Venezuela (481 cases) and Guyana (97 cases) represented 80% of the *P. falciparum* registered in the city (722 cases) (1). Pacaraima is located in the north of the state (04º25′51'' N; 61º08′45'' W), in a mountainous region, above 1000 m altitude with an estimated population of 17,401 inhabitants, including 5838 indigenous peoples of three ethnicities (Makuxi, Taurepang, Wapixana). This municipality presents an intense movement of people between Pacaraima and Santa Elena do Uairén. The imported malaria in this municipality is almost exclusively from Venezuela. Migration fluctuates according to the treatment offered in Santa Elena do Uairén, a border town with Pacaraima, or according to local political tensions; for example, in 2019, several conflicts in the region caused the closure of the border, with a decrease in the number of imported malaria cases from 2137 cases in 2018 to 268 cases in 2019. However, there has been an increase in autochthonous malaria cases in the indigenous communities (1577 cases) [1]. São João da Baliza is located in the south of the state (0º57′03'' N; 59º54′39'' W), bordering Amazonas state, at a distance of 326 km from Boa Vista. The municipality is sparsely populated, with an estimated population of 8,201. In 2019, São João da Baliza reported 634 malaria cases; 461 were autochthonous, of which 310 were acquired in the urban area of the municipality, equivalent to 66.2%, against 151 in the rural area. Imported malaria cases were low, with only two cases from Venezuela and French Guiana [1].

**Fig. 2:**
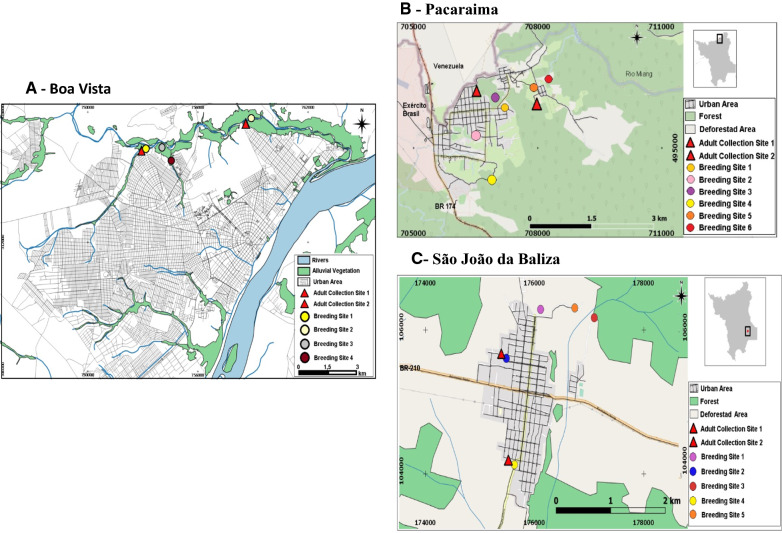
Location of larval habitats, adult collection sites, and surrounding vegetation in Boa Vista (**A**), Pacaraima (**B**), and São João da Baliza (**C**)

Study design and larval habitat collection larval and adult mosquitoes were collected every two months from January 2017 to October 2018. In each municipality, four to six mosquito larval habitats were selected and sampled for larvae and pupae, and two residences near the larval habitats were selected for adult mosquito collection (Fig. 2). The larval habitats were selected based on their proximity to houses where autochthonous cases occurred the year prior to the study and were positive for *Anopheles* spp. larvae in a preliminary survey conducted in October 2016. For each larval habitat, the following physicochemical and environmental parameters were determined: habitat type, size, water temperature, water pH, degree of water turbidity, sun exposure, stream current, and vegetation. GPS estimated the distance from the larval habitat to the nearest house. Millimetric rods were used to measure the depth and size of the water bodies. The pH was determined using coloured dipsticks (MerckTM), and sun exposure and stream currents were visually estimated. Each larval habitat was identified by a number and georeferenced to determine its location (Fig. 2). In Boa Vista, the four larval habitats presented surrounding vegetation, and their geographical landscapes were characterized by the existence of savannah with and without gallery forests. In Pacaraima, 83% of the six larval habitats were located in the mountainous region of an ombrophilous forest. In São João da Baliza, 80% of the six larval habitats were surrounded by lowland tropical rainforest vegetation in a deforested area [38, 39].

### Larval surveys

Larval surveys were performed on the same days as adult collections, from 07:00 to 13:00 h and in the afternoon, when necessary. Larval collection was performed using a 350-mL manual dipper (BioQuip, Ranch Dominguez, CA, USA) following a standardized larval sampling methodology recommendation of the Health Surveillance Secretariat of the Ministry of Health. The larvae and pupae collected were transferred to plastic tubes labelled with the larval habitat code, location, date, and collector's name. Larvae and pupae were either placed in 80% ethanol or reared in the laboratory to fourth instars or to adults, respectively, for identification using in Consoli and Lourenço-de-Oliveira dichotomous key for anopheline larvae [40].

### Adult survey

Adult mosquitoes were collected in January, April, July and October of 2017 and 2018, comprising dry (November to April) and rainy (May to October) seasons. In each municipality, the collections were performed simultaneously at two sites, for three consecutive days at sunset from 18:00 to 21:00 and compared in the intradomicile, peridomicile and extradomicile areas. Sampling efforts throughout the studied period consisted of 480 h in each site: 144 h in the intradomicile (Nasci aspirator), 144 h in the peridomicile (MosqTent), and 192 h in the extradomicile (96 h with MosqTent and 96 h with Shannon).

In the intradomicile, mosquitoes were collected at rest on the internal walls and furniture, inside the residence, using a Nasci aspirator [41]. Peridomicile collections were performed approximately 15 m from the house using a protected human-baited mosquito trap (MosqTent) [42]. Extradomicile collections were carried > 15 m from the residence and the main larval habitat using MosqTent. When a horse was available, a baited Shannon trap was also used in the extradomicile concomitantly with the MosqTent collections. In order to assess the peak of haematophagy, collections were also performed in the intradomicile and extradomicile, for a 12-h period, from 18:00 to 06:00, in the months of April (transition from dry to rainy season) and October (transition from rainy to dry season) of 2017 and 2018. All data in relation to each collection (temperature, humidity, wind, rain) were recorded. Mosquitoes were collected using a manual Castro aspirator, placed in paper cages and transported to the Laboratory of the State Entomology Centre in an appropriate Styrofoam box. The specimens were then euthanized with ethyl acetate. Individuals were transferred to plastic tubes (Eppendorf type) and kept in boxes containing hermetically sealed silica gels [40].

Species were morphologically identified using dichotomous keys for the Neotropical *Anopheles* species. Members of the *An. albitarsis*, *An. nuneztovari* and *An. triannulatus* complexes were not identified in this study; these species are referred to as sensu lato. Daily and monthly rainfall data were obtained from the National Water Agency (Agência Nacional de Águas, ANA).

### Geographical location of studied sites

The geographic locations of larval habitats and adult collection sites were georeferenced in the Universal Transverse Mercator (UTM) coordinate system using the World Geodetic System 84 (WGS84). All geographic data were processed in the Landscape Metrics Laboratory of the Department of Geography of the Federal University of Roraima (UFRR), using satellite images and a cartographic basis of the municipalities. The data were processed in the QGIS 2.14.17 software to generate the maps such as those previously described [8].

### Statistical analysis

Statistical analyses were performed using GraphPad InStat, version 3 (GraphPad Software, San Diego, CA, USA) and R version 4.0.5. Comparisons between mean temperature and pH were performed using the Wilcox test. To evaluate the association of environmental factors with the occurrence of species in larval habitats, univariate logistic models was used with species as the outcome. In particular, the environmental factors (habitat type, area, water turbidity, water current, water pH, water temperature, house distance, sunlight exposure, and debris) were used as independent variables. The associations between species and environmental variables were presented as odds ratios (ORs) and their 95% confidence intervals. Differences between the two non-reference categories were related to reference categories.

## Results

### Characteristics of larval habitats

*Anopheles* spp*.* larvae were found in all 15 permanent larval habitats in the studied municipalities: four sites in Boa Vista, six in Pacaraima, and five in São João da Baliza. Table 1 shows the main characteristics of each larval habitat and larvae of *Anopheles* spp. were found in all four types of water collection. Streams were widely distributed (66.6%) in the study area, followed by dams (13.3%), ponds (13.3%) and lakes (6.6%). In Boa Vista, the types of water collections were streams and lakes; in Pacaraima, streams, ponds and fishponds, and São João da Baliza, streams and dams. Furthermore, 26.7% of the habitats had a surface area of < 150 sq m, while 40% were > 150 < 300 sq m and 33.3% had a surface area greater than 300 sq m. The majority of the larval habitats in Boa Vista were located at a distance > 15 m from houses, while in Pacaraima and São João da Baliza, they were < 15 m. *Anopheles* spp. larvae were found in both transparent (60%) and turbid (40%) water habitats. In most larval habitats, the water current was slow-moving (53.3%) or stagnant (26.7%), and the temperature and pH were distinct in the habitats sampled. In the municipality of Pacaraima, the larval habitats presented a lower water temperature (20.48 ± 1.0) than in Boa Vista (25.5 ± 3.2; p < 0.0001) and São João da Baliza (26.4 ± 0.95; p < 0.01), and a higher pH (6.0 ± 0.75) when compared to Boa Vista (4.1 ± 1.15; p < 0.01). This study further observed that 53.3 and 33.3% of the habitats were partially shaded and shaded, respectively. Half of the larval habitats in Pacaraima were shaded, while in Boa Vista and São João da Baliza the majority were partially shaded. Only two larval habitats were in full sun, one of which was in Pacaraima and the other in São João da Baliza. Marginal vegetation was present in all larval habitats, while emerging vegetation was present in 73.3%, underwater in 40%, and floating vegetation in 33.3%. Debris such as tree trunks, leaves and roots, were found in all water bodies except in one fishpond in São João da Baliza.

**Table 1:** Characteristics of the larval habitats in Boa Vista, Pacaraima and São João da Baliza

Municipalities	Characteristics	Larval Habitats					
		Site 1	Site 2	Site 3	Site 4	Site 5	Site 6
Boa Vista	Habitat type	Stream	Lake	Stream	Stream	–	–
	Area (sq m)	> 300	> 150 ≤ 300	> 300	> 150 ≤ 300	–	–
	Water turbidity	Turbid	Transparent	Turbid	Transparent	–	–
	Water current	Stagnant	Stagnant	Slow	Slow	–	–
	Water temperature °C (M ± SD)	23.2 ± 10.5	22.3 ± 9.9	28.7 ± 14.9	27.8 ± 14.5	–	–
	Water pH (M ± SD)	5.4 ± 2.4	4.7 ± 2.4	2.9 ± 3.1	3.4 ± 2.9	–	–
	Distance from houses (m)	> 15	> 15	> 15	≤ 8	–	–
	Sunlight exposure	Partly shaded	Partly shaded	Partly shaded	Shaded	–	–
	Debris	Trunk/Roots/Leaves	Trunk/Roots/Leaves	Trunk/Roots/Leaves	Trunk/Roots/Leaves	–	–
	Vegetation	Emerging/Marginal	Emerging/Marginal Floating/Underwater	Emerging/Floating Marginal	Floating/Marginal	–	–
Pacaraima	Habitat type	Pond	Stream	Stream	Stream	Fishpond	Stream
	Area (sq m)	≤ 150	> 150 ≤ 300	> 150 ≤ 300	≤ 150	> 300	> 150 ≤ 300
	Water turbidity	Turbid	Transparent	Transparent	Transparent	Turbid	Turbid
	Current water	Slow	Moderate	Slow	Moderate	Stagnant	Slow
	Water temperature °C (M ± SD)	20.9 ± 8.5	20.1 ± 8.1	19.2 ± 8.5	19.5 ± 8.6	22 ± 9.7	21.2 ± 9.4
	Water pH (M ± SD)	5.5 ± 1	6.3 ± 2.8	6.9 ± 0.7	5.4 ± 2.4	7 ± 0.8	5.4 ± 2.2
	Distance from houses (m)	≤ 8	> 8 ≤ 15	≤ 8	> 8 ≤ 15	> 15	> 15
	Sunlight exposure	Partly shaded	Shaded	Shaded	Shaded	Sunny	Partly shaded
	Debris	Trunk/Roots/Leaves	Trunk/Roots/Leaves	Trunk/Roots/Leaves	Trunk/Roots/Leaves	Nothing	Trunk/Roots/ Leaves/Fruits
	Vegetation	Floating/Underwater Marginal	Emerging/Floating Marginal	Emerging/Marginal	Emerging/Marginal	Underwater	Emerging/Marginal Underwater
S.J. Baliza	Habitat type	Dam	Stream	Stream	Stream	Dam	-
	Area (sq m)	> 300	≤ 150	> 300	> 150 ≤ 300	≤ 150	-
	Water turbidity	Turbid	Turbid	Transparent	Turbid	Turbid	-
	Current water	Slow	Slow	Slow	Moderate	Stagnant	-
	Water temperature °C (M ± SD)	27 ± 2.3	25.8 ± 1.1	25.1 ± 0.4	27.5 ± 1.7	26.6 ± 2.3	-
	Water pH (M ± SD)	5.5 ± 2.3	5.5 ± 2.3	6.5 ± 0.8	5.5 ± 2.3	5.5 ± 3.5	-
	Distance from houses (m)	> 8 ≤ 15	≤ 8	≤ 8	> 8 ≤ 15	> 15	-
	Sunlight exposure	Partly shaded	Partly shaded	Partly shaded	Shaded	Sunny	-
	Debris	Trunk/Roots/Leaves	Trunk/Roots/Leaves	Trunk/Roots/Leaves	Trunk/Roots/Leaves	Nothing	-
	Vegetation	Emerging/Marginal	Emerging/Marginal	Emerging/Marginal	Emerging/Underwater Marginal	Underwater	-

– Not done

### Identification of immature forms of anophelines

A total of 544 *Anopheles* spp. specimens were obtained from 15 larval habitats, and the relative abundance of each larval species encountered is presented in Table 2. A total of 12 species were identified from 209 late-instar larvae (third and fourth stages). However, 335 *Anopheles* spp larvae were not identified at the species level and considered for statistical analysis, mainly because they were early instar larvae (first and second stages) or were damaged. Collections of the immature forms showed that *An. albitarsis s.l.*, *An. nuneztovari s.l.*, *An. triannulatus s.l.*, and *Anopheles peryassui* were collected from larval habitats from all municipalities while *An. darlingi*, *Anopheles matogrossensis* and *Anopheles oswaldoi s.l.* were only observed in Boa Vista and São João da Baliza. *Anopheles evansae* was present in Boa Vista and Pacaraima, *Anopheles mediopunctatus* and *Anopheles argyritarsis* only in Pacaraima and *Chagasia bonneae* and *Anopheles strodei* were only found in São João da Baliza.

**Table 2:** The relative abundance of each larvae species collected at larval habitats in the municipalities of Boa Vista, Pacaraima and São João da Baliza

Municipalities	Species	Larval habitats						
		Site 1	Site 2	Site 3	Site 4	Site 5	Site 6	Total
		N (%)	N (%)	N (%)	N (%)	N (%)	N (%)	N (%)
		Stream	Lake	Stream	Stream	–	–	
Boa Vista	*An. darlingi*	13 (65.0)	15 (38.5)	0	0	–	–	28 (44.4)
	*An. albitarsis s.l.*	0	6 (15.4)	0	0	–	–	6 (9.5)
	*An. nuneztovari s.l.*	2 (10.0)	11 (28.2)	4 (100)	0	–	–	17 (27.0)
	*An. mattogrossensis*	4 (20)	0	0	0	–	–	4 (6.3)
	*An. triannulatus s.l.*	1 (5.0)	0	0	0	–	–	1 (1.6)
	*An. evansae*	0	5 (12.8)	0	0	–	–	5 (7.9)
	*An. peryassui*	0	1 (2.6)	0	0	–	–	1 (1.6)
	*An. oswaldoi s.l.*	0	1 (2.6)	0	0	–	–	1 (1.6)
	Total	20 (31.7)	39 (61.9)	4 (6.4)	0	–	–	63(100)
	*Anopheles spp*.*	18	13	3	2	–	–	36
		Pond	Stream	Stream	Stream	Fishpond	Stream	
Pacaraima	*An. albitarsis s.l.*	0	0	6 (100)	0	2 (13.33)	0	8 (24.2)
	*An. nuneztovari s.l.*	1 (100)	0	0	0	0	0	1 (3.0)
	*An. triannulatus s.l.*	0	0	0	0	11 (73.33)	0	11 (33.3)
	*An. evansae*	0	0	0	0	1 (6.66)	0	1 (3.0)
	*An. peryassui*	0	0	0	4 (100)	1 (6.66)	0	5 (15.1)
	*An. mediopunctatus*	0	0	0	0	0	1 (100)	1 (3.0)
	*An. argyritarsis*	0	6 (100)	0	0	0	0	6 (18.2)
	*Total*	1 (3.0)	6 (18.2)	6 (18.2)	4 (12.1)	15 (45.4)	1 (3.0)	33
	*Anopheles spp.	4	41	32	8	58	5	148
		Dam	Stream	Stream	Stream	Dam	-	
S.J. Baliza	*An. darlingi*	8 (38.0)	16 (66.7)	3 (37.5)	31 (86.1)	4 (16.7)	-	62 (62.9)
	*An. albitarsis s.l.*	1 (4.8)	2 (8.3)	0	1 (2.8)	6 (25.0)	-	10 (8.8)
	*An. nuneztovari s.l.*	9 (42.8)	3 (12.5)	0	0	9 (37.5)	-	21 (18.6)
	*An. mattogrossensis*	0	0	1 (12.5)	0	0	-	1 (0.9)
	*An. triannulatus s.l.*	2 (9.5)	1 (4.2)	0	3 (8.3)	3 (12.5)	-	9 (8.0)
	*An. peryassui*	0	0	1 (12.5)	0	0	-	1 (0.9)
	*Chagasia bonneae*	0	0	3 (37.5)	0	0	-	3 (2.6)
	*An. oswaldoi s.l.*	1 (4.8)	2 (8.3)	0	1 (2.8)	1 (4.2)	-	5 (4.4)
	*An. strodei*	0	0	0	0	1 (4.2)	-	1 (0.9)
	*Total*	21 (18.6)	24 (21.2)	8 (7.1)	36 (31.8)	24 (21.2)	-	113
	*Anopheles spp.	13	11	5	17	105	-	151

– Not done

*Anopheles spp.: *Anopheles that* were not identified at the species level, mainly because they were early instar larvae (first and second stages) or were damaged

In Boa Vista, 99 larvae of eight species were collected mainly from two larval habitats; *An. darlingi* (44.4%) was the most abundant species, followed by *An. nuneztovari s.l.* (26.9%) and *An. albitarsis s.l.* (9.5%*)*. During the study period, in the larval habitats 3 and 4, larvae of *An. nuneztovari s.l.* were collected, while at site 4, only a few second stage *Anopheles* larvae were collected. The municipality of São João da Baliza presented the highest number of larvae collected (264) of nine species and *An. darlingi* was present in all five larval habitats, whereas in Boa Vista, this species was present in only two larval habitats. In São João da Baliza, *An. nuneztovari s.l.* (18.6%) and *An. albitarsis s.l.* (8.8%) were also prevalent. Pacaraima showed a total of 148 larvae of seven species collected, and *An. triannulatus s.l.* (33.3%) was the most abundant species, followed by *An. albitarsis s.l.* (24.2%) and *An. argyritarsis* (18.1%). All *An. triannulatus s.l.* were collected from a single larval habitat (5) in a fishpond. Immature forms of *An. darlingi* were present in streams, lakes, and dams with transparent or turbid water, in partially shaded or sunny habitats. However, in these larval habitats, the water had a lower pH and higher water temperature.

### Association between anopheline larvae and environmental factors

The univariate logistic models for the four most frequent *Anopheles* species are summarized in Table 3. *Anopheles darlingi* was more likely to occur in the stream larval habitat (OR = 6.85, p < 0.01) compared with *An. nuneztovari s.l.*, which was found in a dam (OR = 0.16, p < 0.01). *Anopheles triannulatus s.l.* had a preference for fishponds (OR = 40.7, p < 0.01). Larvae of *An. albitarsis s.l.* and *An. darlingi* had a higher chance of being captured in larval habitats with 150–300 sq m of surface area than those larger than 300 sq m (OR = 3.83, p < 0.05, and OR = 2.27, p < 0.05, respectively. *Anopheles darlingi* was also less abundant in stagnant water (OR = 0.08, p < 0.01) and slow currents (OR = 0.12, p < 0.01) than in moderate water currents. The logistic models also showed a higher chance of *An. triannulatus s.l.* to be found in larval habitats > 300 sq m (OR = 0.13, p < 0.01) than those < 150–300 sq m (OR = 0.29, p < 0.05). *Anopheles albitarsis s.l*. had a greater preference for aquatic habitats with transparent waters over turbid water (OR = 0.24, p < 0.01). In contrast, *An. darlingi* and *An. nuneztovari s.l.* occurred in aquatic habitats with both transparent and turbid water. Habitats exposed to the sun were related to the occurrence of *An. albitarsis s.l.* (OR = 2.93, p < 0.05), and *An. triannulatus s.l.* (OR = 15.67, p < 0.01) in comparison to those partially shaded, while *An. darlingi* showed a preference for partially shaded over full sun habitats (OR = 0.1, p < 0.01). *Anopheles darlingi* was more frequently observed in the presence of roots, tree trunks and leaves (OR = 12.58, p < 0.01) than in the absence of debris, while *An. triannulatus s.l.* was less likely to occur in habitats with debris (OR = 0.08, p < 0.01).

**Table 3:** Univariate model to estimate OR (CI 95%) for the association of environmental factors in larval habitats with the occurrence of *Anopheles* species

Variables		*Anopheles* species			
		*An. albitarsis s.l.*	*An. darlingi*	*An. nuneztovari s.l.*	*An. triannulatus s.l.*
Habitat type	Dam	Ref	Ref	Ref	Ref
	Fishpond	0.91 (0.12;4.46)	0 (0;136.02)	-	40.7 (8.21;322.05)**
	Lake	1.15 (0.34;3.88)	2.21 (0.85;5.89)	0.7 (0.26;1.8)	–
	Pond	–	–	–	–
	Stream	0.58 (0.2;1.75)	6.85 (3.08;16.08)**	0.16 (0.06;0.38)**	0.46 (0.12;1.73)
Area (Sq m)	> 300 m	Ref	Ref	Ref	Ref
	150-300 m	3.83 (1.16;17.35)*	2.27 (1.12;4.67)*	0.48 (0.2;1.15)	0.13 (0.03;0.42)**
	< 150 m	3.82 (1.03;18.32)	1.07 (0.48;2.36)	1.11 (0.46;2.67)	0.29 (0.08;0.89)*
Water turbidity	Transparent	Ref	Ref	Ref	Ref
	Turbid	0.24 (0.1;0.59)**	1.51 (0.75;3.08)	0.73 (0.33;1.68)	-
Water current	Moderate	Ref	Ref	Ref	Ref
	Stagnant	6.9 (1.3;127.59)	0.08 (0.02;0.23)**	-	2.35 (0.71;10.67)
	Slow	6.51 (1.14;122.92)	0.12 (0.03;0.35)**	-	0.6 (0.11;3.43)
Distance houses	> 15 m	Ref	Ref	Ref	Ref
	15-8 m	0.2 (0.03;0.74)*	4.19 (2.06;8.85)**	0.46 (0.19;1.04)	0.48 (0.15;1.33)
	< 8 m	1.74 (0.63;4.59)	2.51 (1.11;5.86)*	0.34 (0.09;0.96)	0.15 (0.01;0.82)
Sunlight exposure	Partly shaded	Ref	Ref	Ref	Ref
	Shaded	2.04 (0.68;5.9)	2.42 (1.1;5.71)*	-	1.86 (0.35;8.8)
	Full sun	2.93 (1.01;8.41)*	0.1 (0.03;0.28)**	0.78 (0.32;1.83)	15.67 (5.06;59.78)**
Debris	Nothing	Ref	Ref	Ref	Ref
	Trunk/Roots/Leaves	0.44 (0.17;1.18)	12.58 (4.66;44.06)**	0.8 (0.35;1.96)	0.08 (0.03;0.21)**

Statistically significant (*p < 0.05; and **p < 0.01; – small sample size

### Species distribution of adult anopheline

A total of 1,488 female *Anopheles* from 11 species were collected from all three municipalities (Table 4). Nine species were identified in Boa Vista and *An. albitarsis s.l.* (88.4%) was the predominant species, followed by *An. darlingi* (6.9%) and *An. peryassui* (3%). Among the seven species collected from São João da Baliza, *An. darlingi* (85.6%) was the most predominant species, followed by *An. albitarsis s.l.* (9.3%), and *An. nuneztovari s.l.* (3.9%). Pacaraima showed lower vector density than the municipalities of Boa Vista, and of the six species identified, the most abundant was *An. braziliensis* (64.6%), followed by *An. peryassui* (18.7%) and *An. albitarsis s.l.* (8.3%). In all three municipalities *An. albitarsis s.l., An. darlingi* and *An. nuneztovari s.l.* were present, whereas *An. matogrossensis* and *An. evansae* were observed only in Boa Vista and *An. triannulatus s.l.* only in São João da Baliza. Overall, the majority of anophelines exhibited greater extradomicile than peridomicile biting preferences. *Anopheles darlingi* was the only species found in the intradomicile. In Boa Vista and São João da Baliza *An. darlingi* and *An. albitarsis s.l.* were more abundant in the extradomicile than in the peridomicile. However, in Pacaraima and Boa Vista *An. darlingi* and *An. albitarsis s.l.* were also collected in the Shannon trap. In Pacaraima, *An. braziliensis* was captured in the peridomicile (33.8%), and in the extradomicile by MosqTent (29%) and by the Shannon trap collection (37.1%).

**Table 4:** Absolute number and relative frequency of adult anopheline species collected in the intradomicile, peridomicile and extradomicile by MosqTent (protected human baited) and Shannon trap (horse baited) from January 2017 to October 2018 in the municipalities of Boa Vista, Pacaraima and São João da Baliza

Municipalities	Species	Sampling environments				Total
		Intradomicile	Peridomicile	Extradomicile		
				Mosquitent	Shannon	
		N(%)	N(%)	N(%)	N(%)	N(%)
Boa Vista	*An. darlingi*	0	20(27.4)	42(57.5)	11(15.1)	73 (6.9)
	*An. albitarsis s.l.*	0	190(20.4)	669(71.9)	71(7.6)	930 (88.4)
	*An. braziliensis*	0	0	8(100)	0	8 (0.8)
	*An. peryassui*	0	3(9.4)	28(87.5)	1(3.1)	32 (3.0)
	*An. nuneztovari s.l.*	0	0	3(60.0)	2(40.0)	5 (0.5)
	*An. strodei*	0	0	0	1(100)	1 (0.1)
	*An. mattogrossensis*	0	0	0	1(100)	1 (0.1)
	*An. oswaldoi s.l.*	0	1(100)	0	0	1 (0.1)
	*An. evansae*	0	0	1(100)	0	1 (0.1)
	Total	0	214 (20.3)	751 (71.4)	87 (8.3)	1052
Pacaraima	*An. darlingi*	0	0	0	1(100)	1 (1.0)
	*An. albitarsis s.l.*	0	2(25.0)	1 (12.5)	5(62.5)	8 (8.3)
	*An. braziliensis*	0	21(33.8)	18(29.0)	23(37.1)	62 (64.6)
	*An. peryassui*	0	6(33.3)	1(5.5)	11(61.1)	18 (18.7)
	*An. nuneztovari s.l.*	0	0	1(100)	0	1 (1.04)
	*An. argyritarsis*	0	3(50.0)	1(16.7)	2(33.3)	6 (6.2)
	*Total*	0	32 (33.3)	22 (22.9)	42 (43.8)	96
S.J. Baliza	*An. darlingi*	4(1.39)	108(37.8)	174(60.8)	0	286 (85.6)
	*An. albitarsis s.l.*	0	9(29.0)	22(71.0)	0	31 (9.3)
	*An. nuneztovari s.l.*	0	3(23.1)	9(69.2)	1(7.7)	13 (3.9)
	*An. argyritarsis*	0	1(100)	0	0	1 (0.3)
	*An. strodei*	0	0	1(100)	0	1 (0.3)
	*An. oswaldoi s.l.*	0	0	1(100)	0	1 (0.3)
	*An. triannulatus s.l.*	0	1(100)	0	0	1 (0.3)
	*Total*	4 (1.2)	122 (36.5)	207 (62.0)	1 (0.3)	334

### Seasonal variation of *Anopheles* in each municipality

**Fig. 3:**
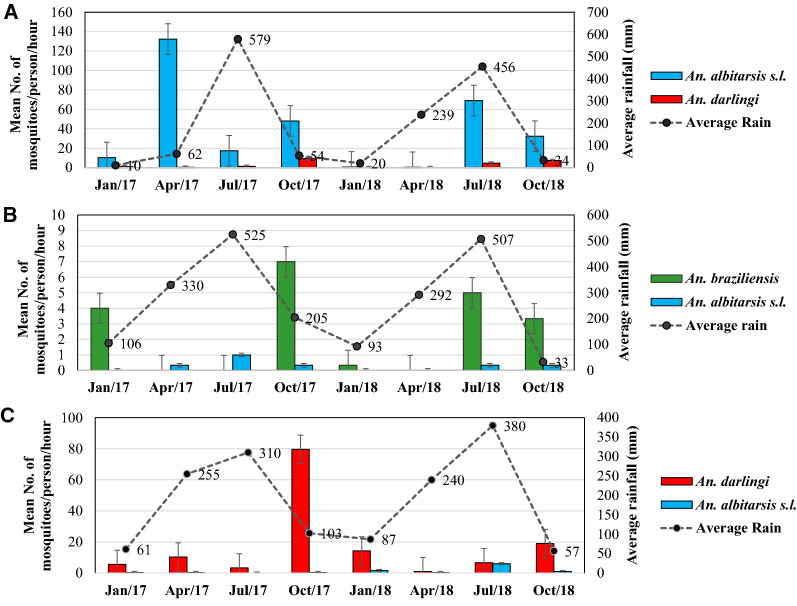
Seasonal dynamics of the most abundant anopheline species in Boa Vista (**A**), Pacaraima (**B**) and São João da Baliza (**C**)

The longitudinal study showed variation in the abundance of *Anopheles* species according to the period studied (Fig. 3). The collection each month represents the mean number of *Anopheles* captured in that month per collector per hour (4 h of capture for three consecutive days) for every two months in each locality.

The rainy season in the state of Roraima occurs between April and September. Even so, in Boa Vista this was the period with the greatest abundance of *Anopheles*. In Pacaraima and São João da Baliza these species were more frequent in the driest months. In Boa Vista (Fig. 3A) *An. albitarsis s.l.* had the highest density during the studied months, with the greatest frequency observed in April in the dry season. The month of July for both years (2017 and 2018) had the greatest rainfall, and a large number of *An. albitarsis s.l.* were captured in 2018. *Anopheles darlingi* was not abundant during the study months but showed a population increase in October at the end of the rainy season.

In Pacaraima (Fig. 3B), *An. braziliensis* was the most abundant species collected in higher numbers in October, two months after the high record for rain precipitation, while *An. albitarsis s.l.* were collected in fewer numbers during most of the months of collection. In São João da Baliza (Fig. 3C), *An. darlingi* was predominant throughout the year, and this species was more abundant in October, whereas *An. albitarsis s.l.* was also collected in every month but in lower numbers.

The dispersion of *Anophele*s by average rainfall intensity and temperature showed that *An. darlingi* was predominant when the temperature was above 24ºC and in the periods with less rainfall, while *An. albitarsis s.l.* prevailed during periods of rain, with temperatures above 23ºC. *Anopheles braziliensis* was collected at lower temperatures (18ºC and 25ºC) during both the dry and rainy periods. *Anopheles peryassui* was the vector that was intermediate to all these periods and was not captured in the extremely dry period (Additional file 1: Fig. S1).

### Biting activity and human bite rate

**Fig. 4:**
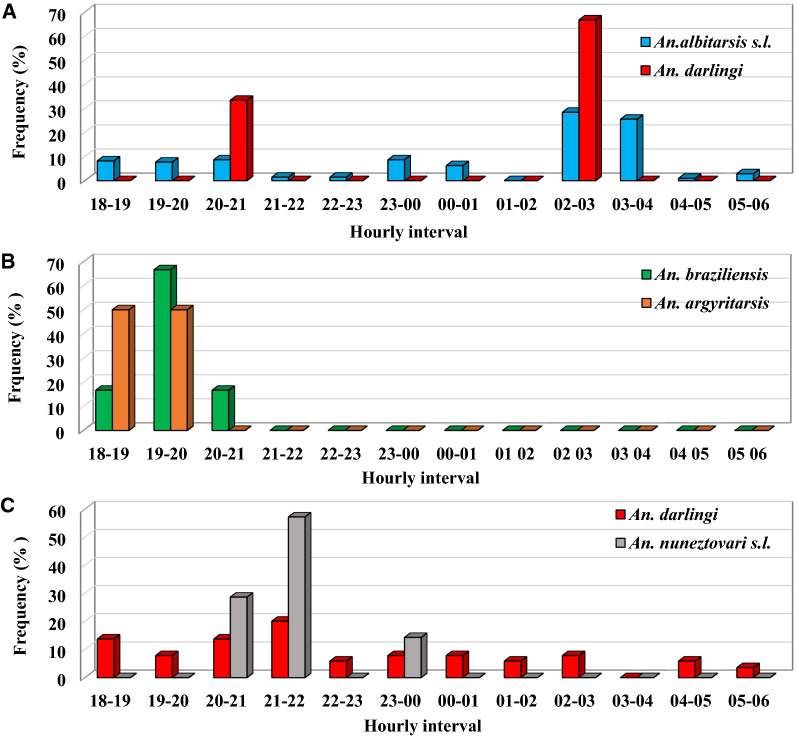
Frequency by time of capture (from 12-h collections) of the main vectors collected in Boa Vista (**A**), Pacaraima (**B**) and São João da Baliza (**C**)

Four 12-h collections were conducted in each municipality simultaneously in the intradomicile and peridomicile to analyse biting activity. Although all collections were performed in both environments, no samples were collected in the intradomicile. Figure 4 shows the frequency (%) of the two more frequent *Anopheles* species in each locality occurring in the peridomicile at hourly intervals. Variability in biting times was observed among species and municipalities. In Boa Vista, *An. albitarsis s.l.*, the most abundant species, presented biting activity at dusk and throughout the night, with most of the activity between 02:00 and 04:00. *Anopheles darlingi* presented biting activity at 20:00–21:00 and throughout the night, but in lower numbers with the majority between 20:00 and 21:00 and 02:00–3:00, with only one specimen of *An. peryassui* captured at 20:00–21:00. In Pacaraima, the most frequently occurring species was *An. braziliensis,* with a significant peak at 19:00–20:00. *Anopheles argyritarsis* was caught between 18:00 and 19:00 and 19:00–20:00, and *An. peryassui* at 18:00–19:00. In the municipality of São João da Baliza, *An. darlingi* exhibited a pronounced crepuscular biting activity with an early peak at 21:00–22:00. However, they were also collected throughout the night until 06:00. *Anopheles nuneztovari* was the second-most common species, occurring from 20:00 to 21:00, 21:00 to 22:00 and from 23:00 to 00:00, while *An. albitarsis s.l.* occurred in higher numbers at 05:00 to 06:00.

The human biting rate (HBR) was estimated for the most common species collected in each municipality using the following calculation: the total number of anophelines captured during the 4-h collection (16:00 to 21:00) divided by the total number of collection days and the number of collectors. The data are presented in Table 5. Among all species collected in Boa Vista, *An. albitarsis s.l.* showed the highest rate in the extradomicile (10.8 bites per night) followed by 3.8 in the peridomicile. The presence of *An. darlingi* predominates with a low HBR in the peridomicile (0.3 bites) and in the extradomicile (0.8 bites). In Pacaraima, HBR was low in both the peridomicile (0.26) and extradomicile (0.22) for *An. braziliensis*. In the municipality of São João da Baliza, *An. darlingi* presented higher HBR in the peridomicile (1.07) and in the extradomicile (1.64) than in Boa Vista while *An. albitarsis s.l.* had a very low HBR. Moreover, São João da Baliza was the only municipality where *An. darlingi* was collected in the intradomicile (three specimens at 19:00–20:00 and one at 20:00–21:00).

**Table 5:** Human biting rate per night by species in the peridomicile (PD) and extradomicile (ED) for each municipality

Municipalities	Species	PD	ED
Boa Vista	*An. albitarsis s.l.*	3.8	10.8
	*An. darlingi*	0.3	0.8
Pacaraima	*An. braziliensis*	0.26	0.22
	*An. argyritarsis*	0.03	0.01
São João da Baliza	*An. darlingi*	1.07	1.64
	*An. albitarsis s.l.*	0.08	0.22

## Discussion

Malariogenic potential measures the risk of transmission in a given area. It is critical for determining strategies to eliminate and prevent the re-establishment of malaria transmission [43], particularly in areas where the potential of imported malaria is high. As part of an integrated strategy for control and elimination, interventions should be deployed regionally based on detailed knowledge of local vector habitats and distribution. Here, during two years, the larval habitat and adults were sampled to determine the *Anopheles* species composition, seasonal occurrence and distribution of vectors in areas of autochthonous (São João da Baliza) and with high numbers of imported (Boa Vista and Pacaraima) malaria in Roraima. Differences in mosquito distribution and richness among sites may be the most important factors for identifying local variation in malaria transmission. Roraima presents a diverse ecological landscape and malaria transmission in Boa Vista, which is mainly savannah, which differs from that of the Yanomami Indian reserves in the northwestern mountains, which is a region with dense rainforest [24–26, 38, 44].

In all municipalities, the most-encountered larval habitats were streams. *Anopheles darlingi* was the most abundant larval species in São Joao da Baliza and Boa Vista. However, in São João da Baliza, where vegetation consists of a lowland tropical rainforest in a deforested area, *An. darlingi* occurred at all five larval habitats, while in Boa Vista, a savannah vegetation, this species was present in only two larval habitats and in lower numbers. The differences in the ecological environment between Boa Vista and São João da Baliza may account for this [38]. Studies have shown that *An. darlingi* is often associated with low-lying vegetation along forested river margins and deforestation provides favourable conditions for this vector, thereby increasing malaria cases and decreasing scores of the Human Development Index [14, 45–48].

*Anopheles darlingi* has been found in a variety of larval habitats, both natural and man-made, and from large and permanent to small and temporary water bodies [13, 46–48]. In Boa Vista and São João da Baliza, *An. darlingi* were collected mainly in aquatic habitats with transparent water and moderate current with temperatures between 22 °C and 28 °C, in partially shaded areas with surrounding vegetation. According to Hiwat and Bretas [14], the immature stages of *An. darlingi* develop in aquatic habitats partial shaded, with temperature and pH varying, respectively, between 20 and 28 ℃, and 6.5 and 7 [14].

In Pacaraima, a mountainous region of an ombrophilous forest, *An. darlingi* larvae were not observed but one adult mosquito was collected. This species is not normally found in areas with high altitudes and in low temperatures [40]. However, recently it was collected at altitudes above 800 m in Venezuela, close to the border of Roraima [13]. Instead, *An. triannulatus s.l.* was the most abundant species collected in a single fishpond exposed to the sun that was located in a horticultural area and also in dams and streams in savannah and tropical forest larval habitats. *Anopheles triannulatus* *s.l.* seems to be a generalist as it demonstrated both widespread distribution and little if any environmental constraints [20, 49, 50]. This species appears to play an important role in malaria transmission in the states of Amapá, Amazonas, Pará and Peru [21, 23, 51, 52].

*Anopheles albitarsis s.l., An. nuneztovari s.l., An. triannulatus s.l., An. oswaldoi s.l.,* and *An. peryassui* were found in all municipalities but in low numbers. In addition to the composition of anopheline larval species, a total of 1488 adult female *Anopheles* of 11 species were collected from all three municipalities. In São João da Baliza, where autochthonous malaria occurs, the most abundant species was *An. darlingi* (85.6%), while in Boa Vista, the municipality with a high number of imported cases, *An. albitarsis s.l*. (*88.4*%) was predominant, while *An. braziliensis* (64.6%) was abundant in Pacaraima. Except for *An. peryassui*, all species collected have been implicated as malaria vectors in the Brazilian Amazon [21, 53, 54]. Previous studies in Boa Vista corroborate this report regarding the composition of species found, with a greater abundance of *An. albitarsis s.l.* and a lower density of *An. darlingi* [24–26]. *Anopheles albitarsis s.l*. also had the highest HBR with 10.8 bites in the extradomicile and 3.8 in the peridomicile. However, in the 12-h collection period it presented biting activity throughout the night, with the highest biting peak between 02:00 and 04:00, which differs from that previously reported [24–26]. *Anopheles albitarsis s.l.* has been assigned as an important vector in Para, Roraima, Amapá and Rondonia [23, 27, 54]. Because molecular analysis between members of *An. albitarsis, An. nuneztovari*, *An. triannulatus,* and *An. oswaldoi* complexes were not defined in this study, the presence of *An. janconnae* or other species from this complex could not be confirmed in the studied municipalities. Presently, the only species of the *An. albitarsis* complex identified in the state of Roraima, has been *An*. *janconnae*, considered an important malaria vector in savannah habitat around Boa Vista [26]. Entomological surveillance to identify the component members of these complexes are mandatory to update the geographic distribution, ecological and biological aspects, and its possible role as a regional vector of malaria parasites in Roraima.

In Boa Vista, *An. darlingi* were collected in smaller numbers from 20:00 to 21:00 and 02:00 to 03:00, and its presence emerges with low HBR of 0.3 bites in the peridomicile, and 0.8 bites in the extradomicile. The presence of *An. darlingi,* even at low density in Boa Vista, can significantly contribute to transmission because it is highly anthropophilic and susceptible to *Plasmodium* parasites*.* Minimal biting indices could be sufficient to maintain the transmission cycle in Boa Vista [24, 51].

In São João da Baliza, *An. darlingi* was the most abundant species throughout the year, with a pronounced crepuscular biting activity in the peridomicile and extradomicile with early peak at 21:00–22:00 and throughout the night until early morning. Tadei et al. also found similar biting activity, whereas in Amapá this species showed a wide range of blood-feeding pattern [53–55]. In Venezuela, this mosquito bites throughout the night, with minor peaks at 23:00–00:00 and 03:00–04:00 [56]. The time of biting activity of anophelines depends on several factors, such as species density, seasonality and host availability [53, 57].

Regarding seasonality, there was no significant correlation between abundance (of the species) and rainfall or the abundance of each species with locality or rainfall (p > 0.05). The lack of correlation between anopheline species and rainfall could be due to the variability in mosquito abundance among localities*.* In Boa Vista, the highest density of *An. albitarsis s.l.* occurred during the dry season but it was also collected in the period of high rainfall in 2018. *Anopheles darlingi* showed a peak after the rainy season (transition from rainy to dry season), in both years of collection, and a slightly lower peak in the middle of the rainy season, only in 2018. In Rondonia, genetic differences between the populations of *An. darlingi* were characterized by microsatellites with distinct seasonal patterns [58], one of which, population A, presented the highest density at the end of the rainy season, and was characterized as a less abundant population in interior regions than in riparian areas. Another sub-population, population B, peaked at the beginning of the rainy season and was more abundant in the interior.

The HBR per night for *An. darlingi* was also considered high in this municipality with 1.64 bites in the extradomicile and 1.07 in the peridomicile. This likely contributes to transmission in this municipality. Heterogeneous biting behaviour of *An. darlingi* in terms of blood feeding inside and outside houses and variations in the peak time of biting have been shown by numerous studies carried out across the Amazon and in Venezuela and French Guiana [15, 40, 47, 51, 56, 59, 60]. This behaviour is considered the main cause of the ineffectiveness of insecticide sprayed inside houses [61, 62]. One of the control measures used in the malaria control programme in Roraima is indoor residual spraying, which may explain the exophilic behaviour of this species in the studied area.

In addition to the presence of *An. darlingi*, *An. nuneztovari s.l*. and *An. albitarsis s.l.* were observed in the peridomicile and the extradomicile, but in low numbers compared to that of *An. darlingi*; *An. nuneztovari s.l.* was the second-most abundant species in São João da Baliza. This species is considered a primary malaria vector in Venezuela, Peru and Colombia [13, 63–65]. In Brazil, *An. nuneztovari* *s.l.* has been found naturally infected with *P. vivax and P. falciparum* in the Amazonian region and it has been incriminated as local vector in Amapá [21, 35, 53, 54]. However, most of Brazilian populations of *An. nuneztovari s.l.* are predominantly zoophlilic and seem not to sustain malaria transmission in the absence of the primary vector *An. darlingi* [15, 66]. In the urban areas of Roraima, studies have shown a low population density and a low number of infectious *An. nuneztovari* *s.l.* [25].

The abundance of *An. darlingi* throughout the year in São João da Baliza, along with their propensity to seek hosts throughout the night, and their ability to adapt host-seeking behaviour to local environments contributes to their impact as the most important vector in this municipality. In contrast in Pacaraima, since *An. braziliensis* was the predominant species, most adults were collected in the Shannon trap, where a horse was used for attraction. *Anopheles braziliensis* is present in most Amazonian states and is considered a secondary vector because it is primarily zoophilic and exophilic and are rarely involved in malaria transmission [25, 40]. Consoli and Lourenço-de-Oliveira [40], claimed that *An. braziliensis* can be found biting during the day, especially when the host is relatively close to its larval habitat [40]. Importantly, it has been found to be infected with human malaria parasites in the states of Amazonas, Amapá, Rondônia and Roraima [15, 19, 54, 67]. Only one specimen of *An. darlingi* was collected in Pacaraima, but there is a report predicting the presence of this vector in indigenous areas in this municipality, where most of the autochthonous cases occur [1, 68]. Taken together, *An. braziliensis* may play some role in malaria transmission in Pacaraima when at high densities.

## Conclusion

This study showed a diversity of anopheline larvae species and habitats in Boa Vista, Pacaraima and São João da Baliza. *Anopheles darlingi* appeared to be the most important vector in São João da Baliza, the area of autochthonous malaria, and *An. albitarsis s.l.* and *An. braziliensis* in areas of low transmission, although there are increasing reports of imported malaria. Considering the behaviour of the vectors in Boa Vista, Pacaraima and São João da Baliza, interventions such as intradomiciliary spraying will likely be insufficient to reduce malaria transmission. It can be speculated that the local vector assemblage in Boa Vista is sufficient to sustain the disease, and also provides a time buffer to mitigate the effects that imported cases would otherwise have if *An. darlingi* were more prevalent. This circumstance offers an opportunity to reduce the effects of cross-border malaria via early diagnosis and timely treatment. In addition, environmental management of vector larval habitats and health education actions addressing individual and collective forms of prevention are indicated.

## Supplementary Information

**Additional file 1: Fig. S1**. Dispersion graph of anopheline mosquitoes by mean intensity of rain, temperature and species.

## Data Availability

The datasets during and/or analysed during the current study available from the corresponding author on reasonable request.
