# Supplementary figures and images for: Larval habitats, species composition and distribution of malaria vectors in regions with autochthonous and imported malaria in Roraima state, Brazil
Source: Malar J. 2022 Jan 14;21:13. doi: 10.1186/s12936-021-04033-1 (PMC8759267; doi:10.1186/s12936-021-04033-1)

## Slide 1
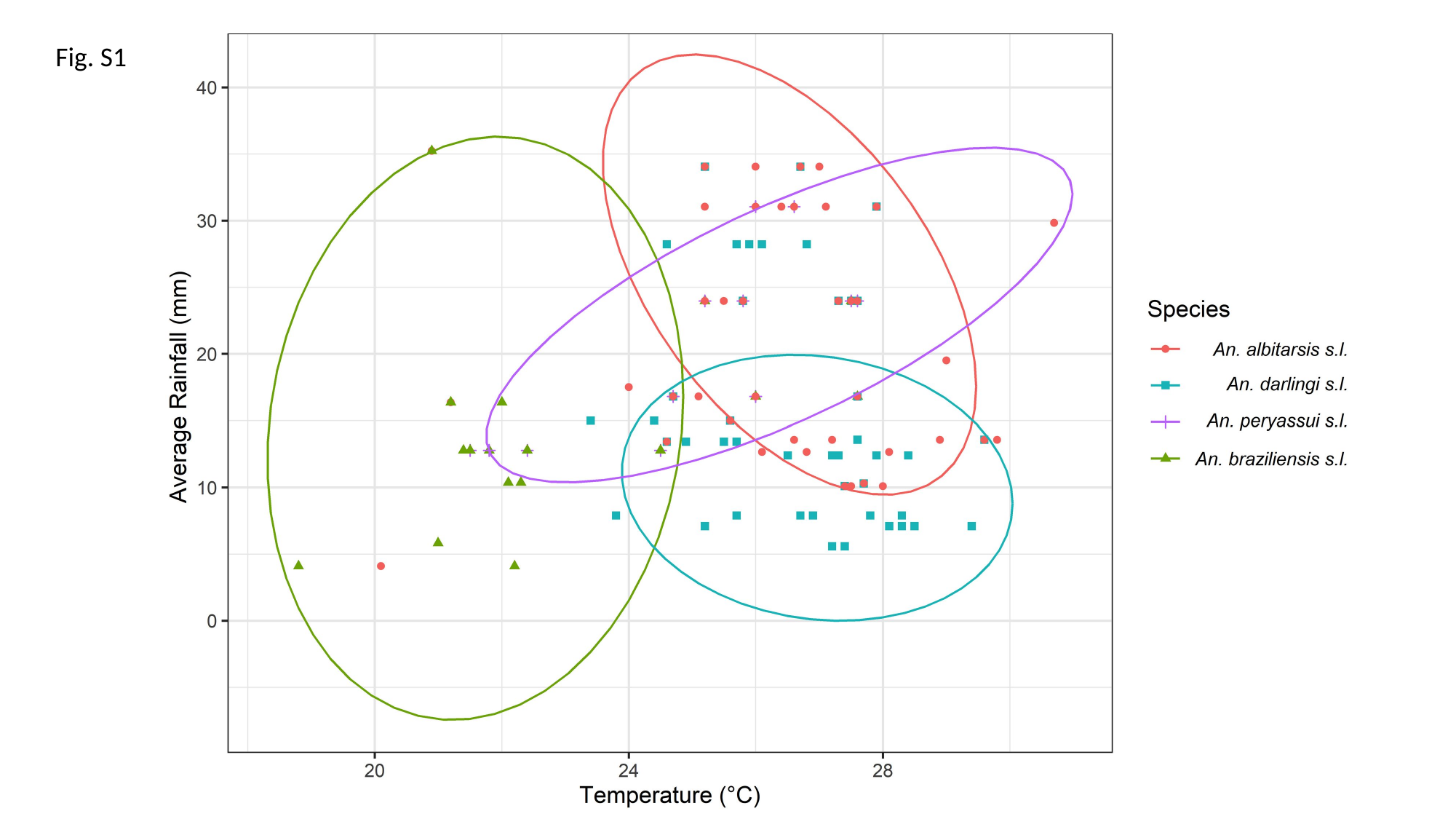

Fig. S1

Supplement: Supplementary file 1 — Additional file 1: Fig. S1. Dispersion graph of anopheline mosquitoes by mean intensity of rain, temperature and species. [file 12936_2021_4033_MOESM1_ESM.pptx]
